# Engineered cord blood megakaryocytes evade killing by allogeneic T-cells for refractory thrombocytopenia

**DOI:** 10.3389/fimmu.2022.1018047

**Published:** 2022-09-20

**Authors:** Bijender Kumar, Vahid Afshar-Kharghan, Mayela Mendt, Robert Sackstein, Mark R. Tanner, Uday Popat, Jeremy Ramdial, May Daher, Juan Jimenez, Rafet Basar, Luciana Melo Garcia, Mayra Shanley, Mecit Kaplan, Xinhai Wan, Vandana Nandivada, Francia Reyes Silva, Vernikka Woods, April Gilbert, Ricardo Gonzalez-Delgado, Sunil Acharya, Paul Lin, Hind Rafei, Pinaki Prosad Banerjee, Elizabeth J. Shpall

**Affiliations:** ^1^ Department of Stem Cell Transplantation and Cellular Therapy, The University of Texas MD Anderson Cancer Center, Houston, TX, United States; ^2^ Section of Benign Hematology, The University of Texas MD Anderson Cancer Center, Houston, TX, United States; ^3^ Department of Translational Medicine, Translational Glycobiology Institute, Herbert Wertheim College of Medicine, Florida International University, Miami, FL, United States

**Keywords:** megakaryocyte, rho-associated coiled coil-containing protein kinase, platelet, cord blood, beta2 microglobin, thrombocytopaenia

## Abstract

The current global platelet supply is often insufficient to meet all the transfusion needs of patients, in particular for those with alloimmune thrombocytopenia. To address this issue, we have developed a strategy employing a combination of approaches to achieve more efficient production of functional megakaryocytes (MKs) and platelets collected from cord blood (CB)-derived CD34+ hematopoietic cells. This strategy is based on *ex-vivo* expansion and differentiation of MKs in the presence of bone marrow niche-mimicking mesenchymal stem cells (MSCs), together with two other key components: (1) To enhance MK polyploidization, we used the potent pharmacological Rho-associated coiled-coil kinase (ROCK) inhibitor, KD045, resulting in liberation of increased numbers of functional platelets both *in-vitro* and *in-vivo*; (2) To evade HLA class I T-cell-driven killing of these expanded MKs, we employed CRISPR-Cas9-mediated β-2 microglobulin (β2M) gene knockout (KO). We found that coculturing with MSCs and MK-lineage-specific cytokines significantly increased MK expansion. This was further increased by ROCK inhibition, which induced MK polyploidization and platelet production. Additionally, *ex-vivo* treatment of MKs with KD045 resulted in significantly higher levels of engraftment and donor chimerism in a mouse model of thrombocytopenia. Finally, β2M KO allowed MKs to evade killing by allogeneic T-cells. Overall, our approaches offer a novel, readily translatable roadmap for producing adult donor-independent platelet products for a variety of clinical indications.

## Introduction

There is an urgent need for a robust and consistently available platelet supply for thrombocytopenic patients. Platelets have a short shelf-life, and hospitals depend on apheresis procedures with adult donors to continuously replenish the supply. The development of an adult donor-independent, off-the-shelf platelet product would alleviate constraints on the platelet inventory and reduce the demand for donors.

Platelets have been successfully generated from human embryonic stem cell (hESC)- and human induced pluripotent stem cell (hiPSC)-derived megakaryocytes (MKs), the cell-type that produces platelets (1–3). However, various concerns, including the expression of oncogenes in hESCs and hiPSCs (1, 4, 5), the usage of non-human serum and feeder cells during culture (3, 6, 7), as well as low platelet yields from *ex-vivo* generated MKs (8) have prevented widespread clinical use of these techniques. However, progress has been made in generating higher yields of platelet-producing MKs in non-human serum- and feeder-free conditions using various techniques, such as spinning embryoid bodies, bioreactors with turbulent flow and shear forces, and culture on gas-permeable surfaces (9–13). These include a study by Ito et al. (10) that used hiPSCs and bioreactors with vertical reciprocal turbulence, which generated 70-80 platelets per MK. Furthermore, using serum-free conditions, Matsunaga et al. (14) generated upwards of 3.4 x 10^4^ platelets per starting human umbilical cord blood (CB) hematopoietic stem cell (HSC), which indicates the potential of generating clinically useful doses of platelets from CB-HSCs. Various small molecule signaling inhibitors and gene expression modifications have also been used to increase MK maturation and produce more functional platelets (10, 13, 15–18). In this study, we developed a novel method using human CB as a source of platelets. We hypothesized that mesenchymal stem cells (MSCs) and MK-lineage growth factors would provide an *ex-vivo*, surrogate hematopoietic niche (19, 20) for robust expansion and differentiation of CD34+ CB-HSCs into MKs. We previously showed that MSCs induce expansion of CB-HSCs to myeloid cells (21). Here, we assessed whether a modified MSC-CB co-culture platform could be used to generate and expand MKs for efficient platelet production.

CB-derived MKs have impaired maturation and release fewer platelets than peripheral blood HSC-derived MKs (22, 23). The downregulation of Rho or Rho-associated coiled-coil-containing kinases (ROCK1 and ROCK2) is critical in MK maturation and leads to endomitosis, polyploidization, and proplatelet formation (10, 24–29). We hypothesized that ROCK inhibition would enhance CB-MK maturation in our MSC-CB co-culture platform, thereby optimizing platelet production. Furthermore, alloimmune platelet transfusion refractoriness (PTR) is a life-threatening condition observed in multiply transfused patients and results in bleeding complications and reduced survival (30, 31). The most frequent immune cause of PTR is the presence of alloantibodies against human leukocyte antigen (HLA) class I epitopes, resulting in rejection of transfused platelets unless HLA-I compatible platelets are transfused (31–36). To address this issue, we utilized CRISPR-Cas9-mediated knockout (KO) of the β-2 microglobulin (β2M) gene to generate HLA-I-deficient CB-HSCs and CB-MKs. We sought to determine whether HLA-I-deficient CB-HSCs could expand *ex-vivo*, differentiate into MKs, evade immune clearance, and generate functional platelets. Overall, this study provides a proof-of-concept for a multifaceted approach to optimize CB-MKs as a consistently available source of off-the-shelf platelets that may be beneficial for alleviating the constraints on the platelet inventory.

## Materials and methods

Additional methods regarding CB processing, platelet collection and quantification, MK and platelet analyses, flow cytometry, western blot, T-cell cytotoxicity, bleeding studies, CRISPR, and animal usage are provided in the supplement.

### MSC co-culture and MK differentiation

CB samples were collected and CD34+ cells were isolated, as described in the supplement, following written informed consent under MD Anderson IRB-approved protocols. CD34+ cells were seeded over 50% confluent BM- or CB-derived MSC monolayers and grown in serum-free good manufacturing practice (GMP) grade SCGM media (Cell Genix, Portsmouth, NH), supplemented with 1% glutamine, penicillin/streptomycin (Thermo Fisher Scientific, Waltham, MA) and recombinant human thrombopoietin (TPO, 50ng/ml), IL-6 (50ng/ml), stem cell factor (SCF, 50ng/ml), IL-3 (5ng/ml), and FLT3-ligand (FLT3-L, 5ng/ml) at 37°C and 5% CO_2_ for the initial 3-4 days. All cytokines were purchased from either Peprotech (East Windsor, NJ) or R&D Systems (Minneapolis, MN). IL-3 and FLT3-L were used for the initial myeloid commitment and were removed after 3 days of culture. Thereafter, the cells were maintained in TPO (50ng/ml), IL-6 (50ng/ml), SCF (25ng/ml), and IL-11 (25ng/ml) until maturation, with media changes every third day. The cells were immunophenotyped at day 10-11 of culture and non-MK-lineage cells were removed using MACS lineage negative selection kits (Miltenyi Biotec, GmbH, Germany). The relatively purified MKs were plated on fresh MSC monolayers and expanded further. ROCK inhibitors (Y27632, 5-10μM, Selleckchem, Houston, TX; KD045, 100nM-10μM, Kadmon Corporation, LLC) were then used for 4-5 days to induce MK polyploidization and maturation in the day 19 CB-MK differentiated product in the absence of MSCs (**Supplementary Figure 1A**).

### CB-MK infusion and chimerism

All animal experiments were performed under MD Anderson Institutional Animal Care and Use Committee-approved protocols. 6-week-old NSG mice were irradiated with 300cGy and infused with CB-MKs at 16-20h post-irradiation. For homing analyses, mice were infused with 5x10^6^ KD045-treated or untreated CB-MKs that were labelled with 2μM carboxyfluorescein succinimidyl ester (CFSE, Thermo Fisher). Mice were sacrificed at 16h post-infusion. The BM, liver, blood, and spleen were harvested and analyzed for the percentage of CFSE+ human CD42+ cells in the non-erythroid (Ter119-) fraction of total live cells by flow cytometry. For BM engraftment studies, CB-MKs were infused in sub-lethally (300cGy) irradiated mice and BM cells were harvested from the femur and tibia by crushing and washing with PBS. Single-cell suspensions were stained with hCD41, hCD42, mCD45, and Ter119 antibodies. The percentage engraftment was determined through counting hCD41+/hCD42+ cells in the Ter119- fraction of the live mice BM cells.

### CRISPR-Cas9-mediated β 2M KO

CRISPR-Cas9 mediated β2M KO was performed on day 3 of MK differentiation. Following KO confirmation, CD34+ cells/MKs were cultured in the standard MK differentiation conditions mentioned earlier. Details regarding the KO are in the supplement.

### Statistics

All statistical analyses were performed using Prism 8 software (GraphPad, San Diego, CA). Two-group comparisons were performed using unpaired *t-*tests unless otherwise noted. Statistically significant p values <0.05 are reported as *p<0.05, **p < 0.01, ***p < 0.001 and ****p < 0.001 The significance test used and sample sizes (n) are reported in each figure legend.

## Results

### CB CD34+ cells undergo robust *ex-vivo* expansion and differentiation in the MSC co-culture system

**Figure 1:**
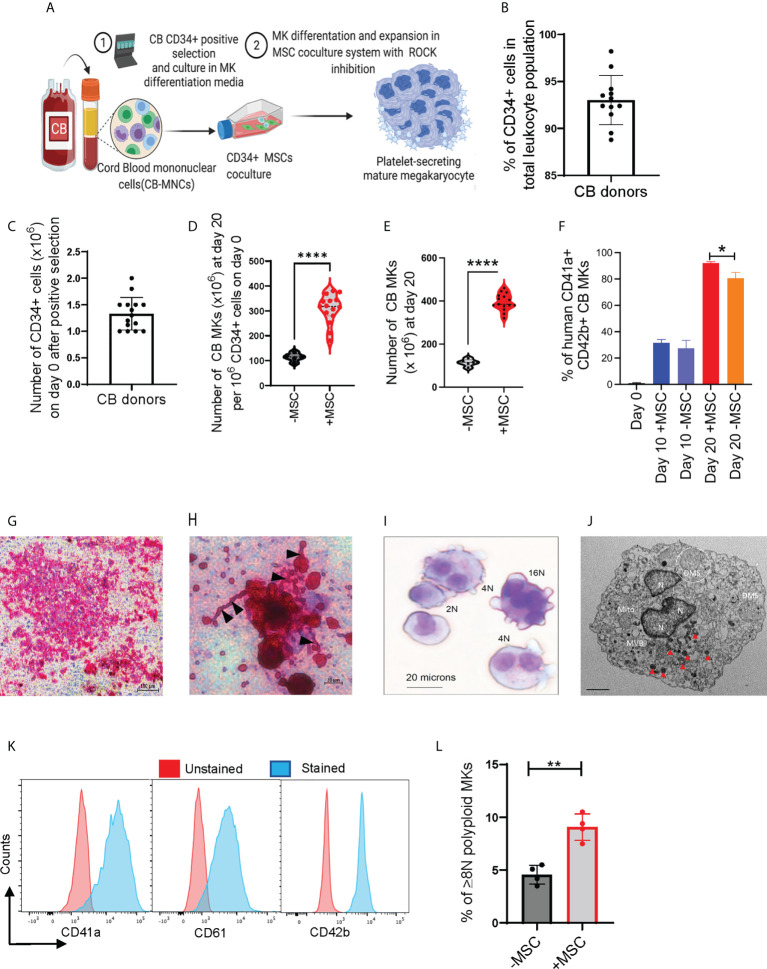
MSCs support HSC expansion and MK terminal differentiation from CB CD34+ cells. **(A)** Schematic of the experimental workflow of MK differentiation and platelet production from CB-derived CD34+ cells in an MSC co-culture system. **(B)** Percentage of CD34+ cells after positive selection amongst multiple cord donors (n=12, each dot represents a different cord). **(C)** Number of CD34+ cells collected per CB unit after positive selection (n=15, each dot represents a different cord). **(D, E)** Violin plots comparing fold-change expansion of CD34+ cells **(D)** and total MKs generated **(E)** with and without MSC support at day 20 (n=15 in MSC co-culture and n=5 without MSCs, ****p < 0.0001). **(F)** hCD41+CD42+ expression pattern in CB-MKs cultured with or without MSCs at day 0, 10, and 20 (n=3-4 CB). **(G)** Human CFU-MK representative image (5X magnification) exhibiting GPIIb/IIIa receptor complex staining in day 12 differentiated CB-MK colonies (scale bar = 100µm). **(H)** Proplatelet formation (black triangles) and released platelets in the day 12 CFU-MK image (scale bar = 20µm). **(I)** Giemsa staining of a day 23 expanded mature CB-MK showing polyploid nuclei. **(J)** Representative transmission electron microscopy (TEM) 5000X magnification image of a CB-MK (Abbreviations: DMS, demarcation membrane system; Mito, mitochondria; MVB, multivesicular bodies; N, nucleus; red arrows, granules; scale bar = 2µm). **(K)** Representative histograms depicting the expression of hCD41a, hCD42b and hCD61 expression (blue) in day 22 MKs compared to unstained controls (red). **(L)** Ploidy of CB-MKs generated in the presence or absence of MSCs and 10µM Y27632, quantified by propidium iodide staining (n=4 with MSCs and 4 without MSCs, **p < 0.01). All statistical analyses completed with unpaired t-tests. * means p<0.05.

We used an *in-vitro* MSC co-culture system with cytokines and pharmacological inhibitors to enrich and differentiate CB-MKs (**Figure 1A** and **Supplementary Figure 1A**). Purified CB-HSCs were characterized as the percentage of CD34+ lineage-cells, with a positivity of 93.0% (n=12 cords) (**Figure 1B**). We collected an average of 1.33 x 10^6^ CD34+ cells per CB unit following positive selection (n=15 cords, **Figure 1C**). CD34+ cells were expanded in serum-free media containing human recombinant TPO and a cocktail of cytokines in liquid culture alone or in co-cultures with MSC support, as described in **Supplementary Figure 1A**. CD34+ cells demonstrated a significantly higher fold-expansion when co-cultured with MSCs at day 20, compared to CD34+ cells cultured alone (308-fold vs. 114-fold, p<0.0001, **Figure 1D**). The MSC co-cultures supported significantly more CD41a+CD42b+ MK expansion and differentiation, compared to the CD34+ cells cultured with cytokines alone. The average number of expanded cells was 395.1 x 10^6^ for those expanded with MSCs and 113.8 x 10^6^ for those expanded without MSCs at day 20 (p<0.0001, n=15 and 5 cords respectively, **Figure 1E**). The percentage of CD41a+CD42b+ CB-MKs expanded with MSCs increased from 31.5% at day 10 to 92.1% (p<0.0001, n=4 cords) on day 20 (**Figure 1F**).

We expanded CB-derived CD34+ cells for 10-12 days with TPO in collagen-based MegaCult-C MK colony-forming (CFU-Meg) assays and visualized their expression of CD41/CD61 (GPIIb/IIIa receptor complex) (**Figure 1G**). We found >50 cells in an MK colony that were actively generating platelets, observed by the presence of proplatelet extensions and demarcation membrane systems (**Figure 1H**) **(** 37). Expanded polyploid MKs on culture day 20 were further verified by the presence of granules and multiple nuclei, as visualized by Giemsa staining (**Figure 1I**) and transmission electron microscopy (TEM) (**Figure 1J** and **Supplementary Figure 2A**). The purity and maturation of the CB-MKs were confirmed by flow cytometry analysis of the MK maturation markers CD41a, CD42b, and CD61 (**Figure 1K**). Finally, MKs generated in the presence of MSCs and 10µM of the ROCK inhibitor Y27632 displayed significantly higher polyploidization (≥8N nuclei) than those generated with 10µM Y27632 but in the absence of MSCs (9.08% with MSCs vs. 4.58% without MSCs, p < 0.01, **Figure 1L**).

### ROCK inhibition increases CB-MK maturation

**Figure 2:**
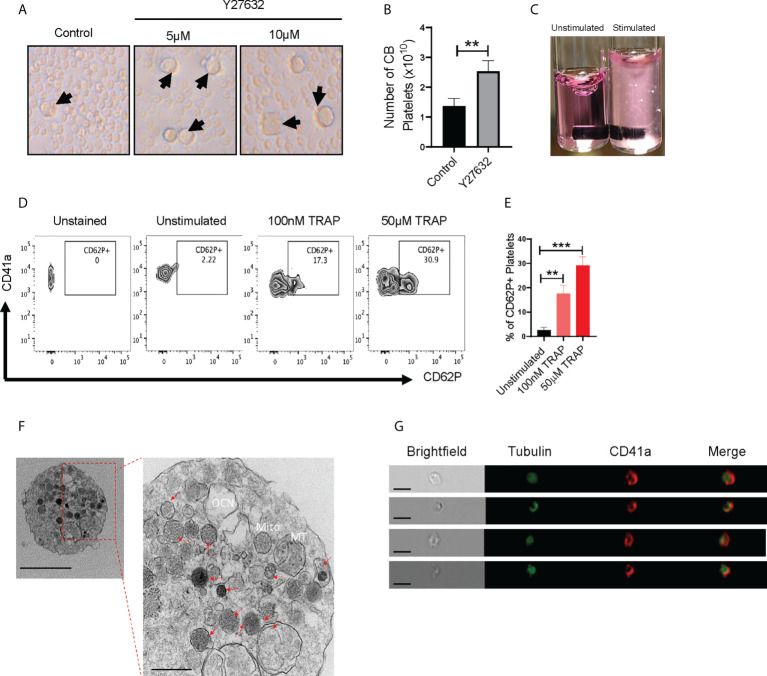
CB-MK-derived platelets are functional and exhibit aggregation characteristics. **(A)** Representative 10X images of differentiating MKs under normal conditions and with 5-10μM Y27632, with examples of larger MKs marked by black arrows. **(B)** Number of secreted platelets from MKs treated with shear stress for 6h and were untreated or pretreated with 10μM Y27632 for 96h (n=3, **p<0.01). **(C)** Representative image of unstimulated and 5μg/ml collagen stimulated platelets in tubes. White triangles indicate aggregating platelets. **(D)** Flow cytometry contour plots depicting CD62P (P-selectin) expression in unstimulated and TRAP-stimulated platelets. **(E)** Frequency of CD62P+ platelets after TRAP stimulation or without stimulation (n=3, **p<0.01 and ***p<0.001). **(F)** Transmission electron microscopy (TEM) 10000X (left, scale bar = 2µm) and 50000X (right, scale bar = 500nm) resolution images of a CB-MK-derived platelet (Abbreviations: Mito, mitochondria; MT, microtubule; OCN, open canalicular network; red arrows, granules). **(G)** Imaging flow cytometry of platelets generated from CB-MKs and assessed for the expression of tubulin and CD41a (scale bar = 7 µm). All statistical analyses completed with unpaired t-tests.

Downregulation of Rho signaling is a critical step in thrombopoiesis (28). ROCK1/2 are directly downstream of Rho, and ROCK inhibition enhances MK maturation and platelet shedding (10, 24–26). We therefore sought to determine whether ROCK inhibitors alter CB-MK differentiation and platelet generation. We treated day 17-19 CB-MKs with 5-10μM of the ROCK inhibitor Y27632 for 4-5 days in the absence of MSCs and observed an increase in the number of large MKs compared to untreated MKs (19μm vs. 10μm average cell size, p=0.02, n=3 cords, **Figure 2A**). This suggests an acceleration in MK maturation with pharmacological ROCK inhibition. We also used shear stress, which increases platelet release from MKs (10, 38), to increase platelet production in day 23 expanded CB-MKs that were treated with or without Y27632. Slight shear stress induced by 6 hour horizontal shaking generated a higher number of platelets from CB-MKs treated with 10μM Y27632, compared to control CB-MKs (2.53 x 10^10^ vs. 1.37 x 10^10^, p=0.0095, **Figure 2B**).

### CB-MKs produce functional platelets

Next, we examined if CB-MK-generated platelets are functional, since earlier studies indicated that CB-derived platelets have impaired aggregation and MSCs may reduce platelet activation, as shown by reduced platelet expression of the activation marker CD62P following MSC co-culture (39–41). Following stimulation with collagen, platelets derived from 10μM Y27632-treated MKs showed a higher amount of visible aggregation than unstimulated platelets (**Figure 2C**). We also stimulated CB-MK-derived platelets with thrombin receptor activating peptide (TRAP). We found a significant dose-dependent increase in CD62P expression in stimulated platelets, compared to unstimulated platelets (2.57% for unstimulated platelets vs. 17.63% for those treated with 100nM TRAP, p=0.0018; 29.13% for those treated with 50μM TRAP, p=0.0003, **Figures 2D, E**). TEM further confirmed the presence of classical surface and intracellular morphology features in CB-derived platelets, including expression of dense, α, and glycogen granules, an open canalicular system, and multiple mitochondria (2-6nm size range, **Figure 2F**, **Supplementary Figures 2B, 3C**). Imaging flow cytometry analysis of platelets derived from CB-MKs indicated co-expression of tubulin with CD41a (**Figure 2G**).

### The highly potent KD045 ROCK inhibitor increases MK platelet production *in-vitro*

**Figure 3:**
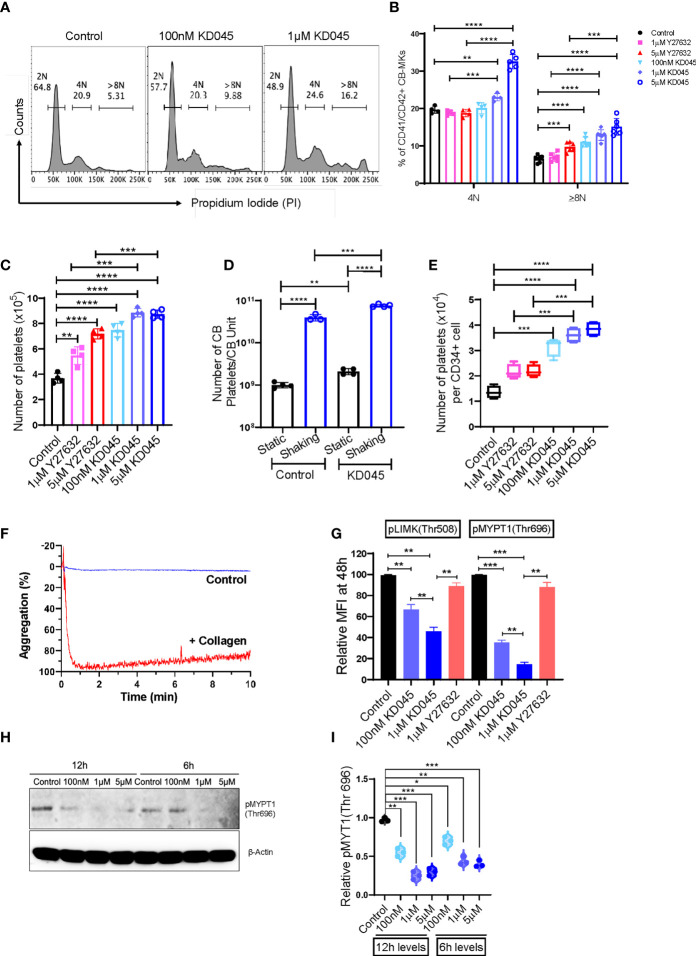
KD045 *ex-vivo* treatment enhances CB-MK polyploidization, reduces ROCK signaling, and stimulates MKs to secrete more functional platelets. **(A)** Histograms showing ploidy analysis by propidium iodide (PI) staining in CD42a+ MKs cultured alone (control) or with 100nM or 1μM KD045 for 96h. **(B)** Percentages of polyploid levels in MKs that were untreated or treated for 96h with Y27632 or KD045 (n=4-6 different cords in at least 3 independent experiments, **p < 0.01, ***p < 0.001 and ****p < 0.0001). **(C)** Numbers of secreted platelets from 1x10^5^ CB-MKs that were untreated or treated with Y27632 or KD045 (n=4 different cords in independent experiments, **p < 0.01, ***p < 0.001 and ****p < 0.0001). **(D)** Number of platelets generated from untreated and KD045-treated day 22 CB-MKs that were subjected to horizontal shaking for 6h prior to platelet quantification. Data are presented as the number of platelets produced per CB unit (n=3-4 per group, **p < 0.01, ***p < 0.001, ****p < 0.0001). **(E)** Number of platelets generated from untreated, Y27632-, and KD045-treated day 22 CB-MKs that were subjected to horizontal shaking for 6h prior to platelet quantification. Data are presented as the number of platelets generated per seeded CD34+ cell on day 0 (n=4 per group, **p < 0.01, ***p < 0.001, ****p < 0.0001). **(F)** Aggregation of unstimulated and collagen-stimulated platelets derived from 100nM KD045-treated CB-MKs. **(G)** Flow cytometry-based pLIMK(Thr508) and pMYPT1(Thr696) intracellular expression, by mean fluorescence intensity (MFI), in untreated, 1μM Y27632, and 100nM-1μM KD045 treated MKs for 48h (n=3, **p < 0.01 and ***p < 0.001 by paired t-tests). **(H, I)** Western blots and normalized relative pMYPT1(Thr696) levels after 6h and 12h treatment with 100nM, 1μM and 5µM of KD045 in day 22 expanded CB-MKs (n=3 independent experiments, *p < 0.05, **p < 0.01, ***p < 0.001 by paired t-tests). All statistical analyses completed with unpaired t-tests unless otherwise noted.

We compared the impact of Y27632 with that of a newly developed and potent second-generation ROCK inhibitor, KD045, on CB-MK maturation and platelet generation. Day 19 CB-MKs were treated with various doses of Y27632 or KD045 for 96h in the absence of MSCs (**Supplementary Figure 1A**). KD045-treated CB-MKs exhibited significantly higher polyploidization (≥8N nuclei) compared to untreated CB-MKs in a dose-dependent manner (6.55% in the control vs. 11.07% for 100nM KD045, p<0.0001; 12.93% for 1μM KD045, p<0.0001; 15.10% for 5μM KD045, p<0.0001, **Figures 3A, B**). KD045-treated CB-MKs also had significantly higher polyploidization than equimolar concentrations of Y27632 (percentage of CB-MKs expressing ≥8N ploidy was 12.93% for 1μM KD045 vs. 7.05% for 1μM Y27632, p<0.0001; 15.10% for 5μM KD045 vs. 9.73% for 5μM Y27632, p=0.0005, **Figure 3B**). KD045 treatment also resulted in a higher proportion of large CB-MKs compared to untreated control CB-MKs, suggesting increased terminal maturation (**Supplementary Figure 4A**).

Furthermore, KD045-treated CB-MKs generated more platelets at day 4 of ROCK inhibitor treatment than untreated CB-MKs. The number of generated platelets from 1 x 10^5^ untreated CB-MKs was approximately 3.68x10^5^ and significantly less than that from 1x10^5^ CB-MKs treated with 100nM KD045 (7.48 x 10^5^, p<0.0001), 1μM KD045 (8.85 x 10^5^, p<0.0001), and 5μM KD045 (8.74 x 10^5^, p<0.0001). KD045-treated CB-MKs also produced significantly more platelets than equimolar concentrations of Y27632 (8.85 x 10^5^ for 1μM KD045 vs. 5.48 x 10^5^ for 1μM Y27632, p<0.001; 8.74 x 10^5^ for 5μM KD045 vs. 7.18 x 10^5^ for 5μM Y27632, p<0.001, **Figure 3C**). In an effort to further optimize the platelet yield from the CB-MKs, we subjected day 22 CB-MKs to horizontal shaking for 6 hours prior to platelet collection and quantification. Horizontal shaking resulted in significantly higher platelet yields compared to those of untreated day 22 CB-MKs (1.01 x 10^9^ platelets per CB unit in static conditions vs. 4.00 x 10^10^ with shaking, p<0.0001), which was further enhanced in day 22 CB-MKs that were pretreated with KD045 (2.10 x 10^9^ platelets per CB unit in static conditions vs. 7.65 x 10^10^ with shaking, p<0.0001, **Figure 3D**). KD045-treatment also resulted in significantly higher platelet yields following 6h horizontal shaking than equimolar concentrations of Y27632 (3.60 x10^4^ for 1µM KD045 vs. 2.16 x 10^4^ for 1µM Y27632, p<0.001; 3.85 x 10^4^ for 5µM KD045 vs. 2.19 x 10^4^ for 5µM Y27632, p<0.001, **Figure 3E**). We also found that platelets generated from 100nM KD045-pretreated CB-MKs had significantly higher aggregation following stimulation with collagen compared to those that were unstimulated (**Figure 3F**). We observed no significant difference in the mean platelet volume of platelets derived from untreated or KD045-pretreated CB-MKs (**Supplementary Figure 4B**).

We also investigated the effect of ROCK inhibition on the expression of molecules downstream of ROCK in CB-MKs. The relative intracellular expression of pMYPT1(Thr696), which is downstream of ROCK, was significantly reduced after 48h treatment with 100nM and 1μM KD045 compared to untreated CB-MKs, as determined by flow cytometry (1.0 for untreated vs. 0.35 for 100nM KD045, p=0.0006; and 0.14 for 1μM KD045, p=0.0002; n=3, **Figure 3G**). Furthermore, 1μM Y27632 was less effective at reducing pMYPT1(Thr696) compared to KD045 (p=0.0019), consistent with our findings regarding increased platelet production by KD045-treated CB-MKs compared to Y27632-treated CB-MKs. These findings were confirmed by western blot, which showed that KD045 nearly abolished pMYPT1(Thr696) expression (1.0 for untreated vs. 0.20 for 1μM KD045, p<0.0001; 0.11 for 5μM KD045, p<0.0001; and 0.08 for 10μM KD045, p<0.0001, n=3 independent experiments, **Supplementary Figures 4C, D**). KD045 also reduced pLIMK(Thr508) expression at 100nM (1.0 vs. 0.67, p=0.0068 in untreated vs. treated cells) and at 1μM (1.0 vs. 0.46, p=0.0012 in untreated vs. treated cells). Equimolar concentrations of KD045 also more potently reduced pLIMK(Thr508) compared to Y27632 (0.46 for 1µM KD045 vs. 0.89 for 1µM Y27632, p=0.002, **Figure 3G**). However, the effect of KD045 on pLIMK(Thr508) expression was smaller than its effect on pMYPT1(Thr696). Short durations of KD045 treatment also induced dose-dependent reductions in pMYPT1(Thr696) (p=0.0013, p=0.0004 and p=0.0008 for 100nM, 1μM and 5μM KD045 respectively at 12h and p=0.012, p=0.0067 and p=0.0004 for 100nM, 1μM and 5μM KD045, respectively at 6h, n=3 independent experiments, **Figures 3H, I**).

We also examined the effect of several cytokines on CB-MK phenotypes. IL-21 reduced the expansion of fully differentiated CB-MKs (p=0.0082, **Supplementary Figure 4E**), while IL-11 and IL-1β increased platelet release by KD045-treated CB-MKs (p=0.004, **Supplementary Figure 4F**). Furthermore, KD045 did not alter the expression of CD41/CD42 in CB-MKs (p=0.14, **Supplementary Figure 4G**). We also found that fewer platelets generated from CB-MKs expressed CD62P compared to peripheral blood platelets (p<0.0001, **Supplementary Figure 4H**). However, CD41/CD42 expression was similar between peripheral blood platelets and CB-MK platelets (p=0.26, **Supplementary Figure 4I**).

### KD045-pretreated human CB-MKs produce platelets in a murine model of thrombocytopenia

**Figure 4:**
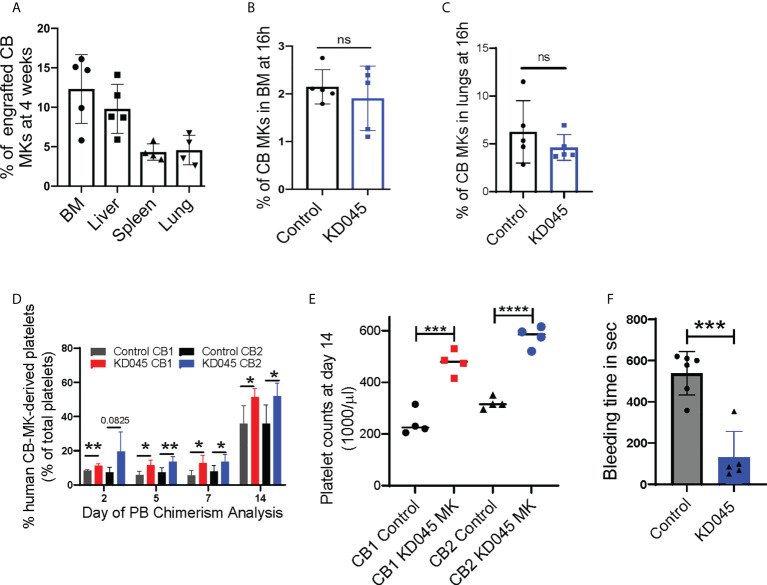
ROCK inhibition increases MK platelet production in an *in-vivo* thrombocytopenia model. **(A)** Percentage CB hCD41a+ hCD42+ chimerism in various niches of sub-lethally irradiated NSG mice at 1-month after infusion of 7 x 10^6^ CB-MKs that were pretreated with KD045 (n=4-5 mice). **(B, C)** Percentages of untreated or KD045 pretreated CFSE+ CB-MKs (in total Ter119- BM live cells) that homed to mice bone marrow (BM) **(B)** and lungs **(C)** at 16h post 5 x 10^6^ CB-MK transfer (n=5 mice per group). **(D)** CB-MK-derived circulating platelet chimerism in mice blood at 2, 5, 7 and 14 days after transfer of 15 x 10^6^ MKs that were either untreated or pretreated with KD045 prior to transfer (n=4-5 mice per group from 2 different CB donors, *p < 0.05 and **p < 0.01). **(E)** Platelet counts in mice blood at 14 days after transfer of 15 x 10^6^ MKs that were either untreated or KD045-pretreated prior to transfer (n=4 mice per group, ***p < 0.001 and ****p < 0.0001). **(F)** Tail bleeding time in seconds of sub-lethally irradiated NSG mice 14 days after infusion of KD045-pretreated MKs, compared to bleeding time of mice that did not receive MKs (n=5-6 mice per group, ***p < 0.001). All statistical analyses completed with unpaired t-tests. "ns" means not significant.

We used a radiation-induced thrombocytopenia model in NSG mice to study the effect of *ex-vivo* ROCK inhibition on donor CB-MKs *in-vivo* (**Supplementary Figure 1C**). Following sublethal exposure of mice to 300cGy radiation, we observed a significant reduction in platelet counts (1200 x 10^9^/L for control vs. 320 x 10^9^/L at day 7 post-radiation, **Supplementary Figure 5**). Within 16-20h following sublethal irradiation, KD045-pretreated human CB-MKs were transferred to the mice by intravenous injection. We evaluated human CB-MK (hCD41+CD42+ vs. mCD45) chimerism at 4 weeks post-radiation by flow cytometry and found that the transferred human CB-MKs could be detected in various organs (**Figure 4A**).

ROCK inhibition alters cytoskeletal proteins (42), which may hinder CB-MK migration and homing. We therefore determined if KD045-treated CB-MKs have impaired BM homing. NSG mice were irradiated with 300cGy and infused with 5 x 10^6^ untreated or 100nM KD045-pretreated CB-MKs. At 16h post-infusion, human CD41+ CB-MK chimerism in the BM revealed that KD045 treatment did not impact the BM homing potential of CB-MKs (2.2% for control CB-MKs vs. 1.9% for KD045 pretreated CB-MKs, **Figure 4B**). Similarly, CB-MK homing to the lung was unaltered by KD045 treatment (6.3% for control CB-MKs vs. 4.6% for KD045 pretreated CB-MKs, **Figure 4C**).

Next, we studied the effect of *ex-vivo* KD045 pretreatment on MKs’ platelet generation capacity *in-vivo*. NSG mice were sub-lethally irradiated and then infused with 15x10^6^ KD045-treated or untreated CB-MKs (**Supplementary Figure 1D**). The percentage of circulating human CD41+ platelets was analyzed (n=2 cord donors, **Supplementary Figure 4J**). We observed a significantly increased percentage of human CD41+ platelets, compared to mouse platelets, in the blood of mice that received KD045-pretreated MKs compared to those that received untreated MKs (n=4-5 mice per group from 2 different CB donors, p=0.03 for CB1 and p=0.03 for CB2 at day 14; **Figure 4D**). The number of platelets circulating in mice at day 14 that received KD045-pretreated MKs was also significantly higher than in mice that received untreated MKs (n=4 mice per group, p<0.001 for CB1 and p<0.0001 for CB2; **Figure 4E**). Mice that did not receive CB-MKs had no detectable human CD41+ platelets (data not shown). In this mouse model of thrombocytopenia, mouse MK-derived platelet counts begin to increase approximately 20 days post-irradiation (**Supplementary Figure 5**). To examine the function of platelets generated from KD045-pretreated CB-MKs, we measured tail vein bleeding time at day 14 post-CB-MK infusion. We found a significant reduction in bleeding time in the recipients of KD045-pretreated CB-MKs (538 seconds for mice that did not receive CB-MKs vs. 130 seconds for mice that received KD045-pretreated CB-MKs, p=0.0002, **Figure 4F**), suggesting that KD045-pretreated CB-MKs and their platelets were functional *in-vivo*.

### CRISPR-Cas9 edited HLA-I deficient CB-MKs exhibit normal expansion and undergo reduced cytotoxic T-cell-induced killing

**Figure 5:**
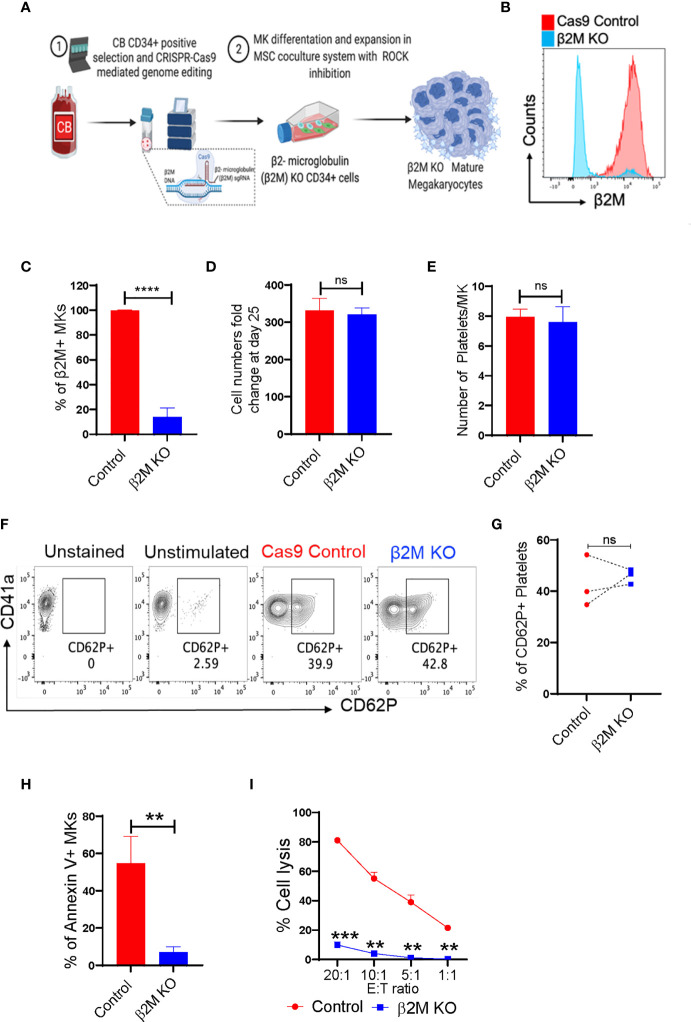
CRISPR-Cas9 engineered β2M KO CB-HSCs/MKs have a similar maturation profile and escape allogenic CD8+ T-cell-mediated killing. **(A)** Schematic of the generation of CRISPR-Cas9 edited β2M KO MKs. **(B)** Histogram of β2M expression in Cas9 control and CRISPR-Cas9 β2M KO CD34+ cells at 72h after electroporation. **(C)** β2M expression of the expanding and differentiating day 6 CB-derived CD34+ cells (n=3, ****p<0.0001). **(D)** Expansion potential comparison between Cas9 control and β2M crRNA+Cas9 treated CB-derived cells (n=4). **(E)** Comparison of the number of platelets secreted per mature MK after 72h culture of day 22, KD045-primed control and β2M KO MKs (n=3, p>0.15). **(F)** Flow cytometry contour plots of CD41a and CD62P expression in control and β2M KO CB-MK-derived platelets. **(G)** Percentage of platelets generated from Cas9 control and β2M KO CB-MKs that expressed CD62P (n=3). **(H)** Apoptosis analysis of Cas9 control and β2M KO day 20 MKs after MK and pre-activated CD8+ T cell co-culture for 4h, followed by annexin V staining of hCD42+CD3- cells (n=3, **p<0.01). **(I)** Cr51 release assay of day 22 expanded Cas9 control and β2M KO CB-MKs co-cultured with pre-activated CD8+ T cells (n=2 cords, **p<0.01, ***p<0.001). Statistical analyses were performed using unpaired t-tests. "ns" means not significant.

β2M plays an important role in HLA class-I-mediated antigen presentation and recognition. Loss of HLA class-I from a cell surface can help that cell evade the immune system. Therefore, selective removal of β2M from CB-MKs could allow their platelets to escape the removal by the immune system in patients with alloimmune PTR. We developed CB-CD34+ HLA class-I-deficient CB-MKs (β2M KO, exon 2) (**Figure 5A** and **Supplementary Figure 1B**) and evaluated the surface expression of β2M. We observed an approximately 90% KO efficiency at day 7 in the differentiating CD34+ cells and early CB-MKs (n=3, p<0.0001, **Figures 5B, C**). On day 25 of culture, including 4 days of KD045 treatment, we observed similar numbers of mature CB-MKs in the Cas9 control and HLA-I (β2M) KO groups (**Figure 5D**). The number of platelets released per CB-MK was similar in both groups (**Figure 5E**), suggesting that β2M ablation does not affect CB-MK proliferation or platelet generation. The levels of CD62P expression were also similar in platelets derived from Cas9 control CB-MKs and β2M KO CB-MKs, indicating similar levels of activation between groups (**Figures 5F, G**).

Direct co-culture of control or β2M KO mature CB-MKs with cytotoxic T-cells was performed for 4h, followed by assessment of CB-MK apoptosis by measuring annexin V. We found a reduced number of annexin V+ cells in β2M KO CB-MKs (54.80% in the control vs. 6.97% in the β2M KO, p=0.005, **Figure 5H** and **Supplementary Figure 6B**), suggesting that β2M KO CB-MKs escape cytotoxic T-cell killing. Through chromium release assays, we found that T-cells killed less β2M KO CB-MKs than control CB-MKs at all effector:target ratios tested (81.2% vs. 9.9% at 20:1, p=0.0005; 55.1% vs. 4.1% at 10:1, p=0.004; 39.0% vs. 1.2% at 5:1, p=0.008; and 21.5 vs. 0.3% at 1:1, p=0.002; **Figure 5I**). Our data are consistent with earlier observations showing that β2M KO iPSC-derived MKs are resistant to HLA mismatched-mediated killing (43, 44).

## Discussion

With an increasing demand for platelets, the global platelet inventory is continually stressed (45, 46). This is exacerbated by the cumbersome logistics of collecting platelets *via* apheresis and the short shelf-life once the platelets are collected. Novel strategies to produce a consistent and robust supply of platelets are urgently needed. Here, we used several methods, including co-culture with MSCs and ROCK inhibition to promote CB-MKs’ maturation and platelet production. In addition, we facilitated immune evasion of MKs by editing their β2M gene expression. Taken together, these modalities may serve as the basis for the development of a new generation of consistently available, off-the-shelf platelets for clinical use in thrombocytopenic patients.

We used CB-derived MKs in this study as CB is a rich source of HSCs and is usually discarded as a waste product but can be readily collected by many world-wide Obstetrical Units and CB banks. These characteristics make CB an ideal source of HSCs from which MKs can be derived. Differentiating MKs requires multiple signals available in the marrow niche (47, 48), including a supportive stromal microenvironment in which MSCs are an integral part (19, 20, 48). We previously developed a co-culture system with human MSCs to expand myeloid cells from CB-HSCs (21). We refined this platform with a cytokine cocktail to expand and differentiate MKs from CB. Our results validate the efficacy of this co-culture approach in that significantly more MKs were derived from a single CB unit expanded in the presence of MSCs compared to those cultured without MSC support.

Cultured CB-MKs also have maturation defects that limit their platelet production (22, 23). ROCK signaling enhances cytokinesis and prevents endomitosis and MK maturation (10, 24–26, 28). Given the availability of highly selective and potent ROCK inhibitors, we used these agents to induce MK maturation. Using a novel ROCK inhibitor, KD045, along with MSC support, we overcame CB-MK maturation defects, allowing their subsequent differentiation into platelet-producing CB-MKs.

We observed that combining KD045 with IL-1β and IL-11 stimulation further enhanced CB-MK production. Earlier studies have shown that IL-11 enhances megakaryopoiesis and IL-1β accelerates platelet generation (49–55). Additional cytokines, including CCL5 and MIP, also increase platelet release (56). Contrary to earlier studies identifying megakaryopoiesis and thrombopoiesis-promoting roles of IL-21 (57), we observed significantly reduced numbers of MKs following IL-21 treatment (**Supplementary Figure 4E**). Although further studies are needed to determine the optimal cytokine milieu for enhancing ROCK inhibition-driven CB-MK maturation, we were able to generate substantial numbers of platelet-producing CB-MKs using our current expansion and ROCK inhibition strategies.

Furthermore, applying shear stress to MKs can increase platelet release (38), and in some experiments (**Figures 2B**, **3D, E**) we used this technique to enhance platelet production *in-vitro*. However, shear stress can damage MKs, which could limit their viability. Thus, in our *in-vivo* studies, we did not apply shear stress to MKs before their transfer to mice. Future studies to determine techniques that allow for the utilization of shear stress to enhance platelet generation without sacrificing MK viability are in progress.

Notably, throughout our *in-vivo* studies, we detected variability in the percentage of CB-MK donor-derived platelets in the peripheral blood of sub-lethally irradiated mice at various time points. This is likely due to inherent differences in the CB cell donors from which the CB-MKs were derived, indicating heterogeneity of MK differentiation potential and platelet production across CB donors. Future studies are in progress to determine the optimal CB donors for MK differentiation. These include examinations of CD34+ cell viability and colony forming unit capability, along with total nucleated cell number and CD34+ cell dose per CB unit. We will compare these characteristics of individual CB units with their capacity to generate CB-MKs and platelets. These findings will enable us to screen CB units to determine which are most suitable for efficient CB-MK production.

Until recently, gene editing of HSC-derived MKs has been challenging and inefficient and as a result, few studies have used CRISPR-Cas9-mediated gene targeting in MKs (58–60). Here, we used CRISPR-Cas9 to knock out (KO) β2M in CB-CD34+ cells, followed by differentiation to mature MKs that evade allogeneic T-cell killing. We anticipate that β2M KO for the abrogation of HLA-I antigen expression in MKs/platelets will be a viable technique for patients with PTR due to their immune evasion. However, loss of HLA-I may leave the CB-MKs vulnerable to killing by natural killer (NK) cells, which recognize and kill cells lacking HLA-I antigens. If this arises, we will determine if overexpression of HLA-E in the CB-MKs protects them from NK-mediated killing. HLA-E inhibits NK cells by binding to inhibitory CD94/NKG2A receptors and its upregulation can reduce NK lysis of cells lacking HLA-I antigens (61, 62). We will utilize this characteristic of HLA-E to safeguard CB-MKs if needed.

One of the critical limiting factors in the use of CD34+ cells to generate platelets has been the yield of MKs and platelets that are generated *in-vitro* (8). In our optimized technique with MSC co-culture, we generated approximately 4 x 10^8^ CB-MKs per CB unit, and with KD045 and horizontal shaking we generated approximately 7.65 x 10^10^ platelets from a CB unit, or 5.8 x 10^4^ platelets per starting CD34+ cell. Yields of MKs and platelets vary widely across previous studies, with upper limits of 2 x 10^5^ MKs and 3.4 x 10^4^ platelets per initial hiPSC or CD34+ HSC placed into culture (63). Our results are in-line with these yields. A unit of transfused platelets typically contains 3-4 x 10^11^ platelets (64). We are further optimizing the production and processing of CB-MKs in our laboratory to produce the maximal number of platelets from the CB-MKs, but it is possible that even a lower number of CB-MKs can provide optimal hemostasis.

Procedures to scale-up our findings and generate CB-MKs and platelets in a good manufacturing practice (GMP)-compliant manner for clinical use are in progress. We will use CD34+ cells from clinically approved, cryopreserved CB units from our FDA-licensed CB bank. Following positive selection and β2M KO, CD34+ cells will be cultured in sequential GMP-compliant, closed bioreactors with MSCs and MK lineage-specific cytokines for 18 days, followed by ROCK inhibition without MSCs for 3 days. After collection and quality control product release the cells will be administered to the patient (**Supplementary Figure 7**). Using this system of CB-MK differentiation, we envision the establishment of a consistent and readily available platelet source that can be given to patients with refractory alloimmune thrombocytopenia. Importantly, our technique will use a closed system, which will reduce potential contamination that can arise with a protocol involving multiple changes in conditions, such as feeder cells, cytokines, and pharmacologic inhibitors. Also, the multistep nature of the process will provide multiple opportunities to increase efficiency with advancing technologies and the use of better reagents once developed.

Overall, we provided a rationale for using multiple modalities to improve CB-MK differentiation and platelet production, including MSC co-culture and the novel ROCK inhibitor KD045 to differentiate CB-MKs and removal of β2M to improve CB-MK immune evasion. These techniques, applied collectively, provide a readily translatable strategy to provide a universal off-the-shelf platelet source to maintain a reliable supply of platelets for vulnerable thrombocytopenic patients.

## Data availability statement

The raw data supporting the conclusions of this article will be made available by the authors, without undue reservation.

## Ethics statement

The studies involving human participants were reviewed and approved by Institutional Review Board, The University of Texas MD Anderson Cancer Center. The patients/participants provided their written informed consent to participate in this study. The animal study was reviewed and approved by Institutional Animal Care and Use Committee, The University of Texas MD Anderson Cancer Center.

## Author contributions

ES, BK and VA-K conceptualized, designed and directed the study. BK performed experiments, interpreted, and analyzed data. MM and RB assisted in generating the engineered MKs. JJ, XW, VW, AG, RC, RD-G assisted with in-vivo experiments. JJ, MM, FRS, MK, and VN assisted in MK generation and functional experiments. PB assisted with the imaging cytometry experiments. MD, LMG, MS, SA, PL, HR, and PB helped with study planning and discussion. ES, BK, MT, VA-K, RS, UP, and JR reviewed all the data, and wrote and edited the manuscript. ES provided financial and administrative support for the study. All authors contributed to the article and approved the submitted version.

## Funding

Funding for this study was provided by National Institutes of Health grants RO1-CA061508-24 and CCSG P30-CA016672 #7690-45.

## Acknowledgments

The authors would like to thank Kiersten Maldonado, Vivien Van and other members of animal facility for their help in the animal experiments. The authors also thank Kenneth Dunner Jr. from the high-resolution electron microscopy facility of MD Anderson for TEM imaging. All graphics and schematics were created with BioRender.com.

## Conflict of interest

ES, BK, MM, VA, RB have filed for a patent MDA20-0115; UTSC.P1219US.P1 “Production of megakaryocytes and platelets in a co-culture system”. ES and RB and the MDACC has an institutional financial conflict of interest with Affimed GmbH and Takeda Pharmaceuticals for the licensing of the technology related to CAR-NK cells. ES participates on Scientific Advisory Board for Bayer, Novartis, Magenta, Adaptimmune, Mesoblast and Axio. According to NIH policies and procedures, the Brigham & Women’s Hospital has assigned intellectual property rights regarding cell surface glycan engineering to RS. RS’s ownership interests were reviewed and are managed by the Brigham & Women’s Hospital and Partners HealthCare in accordance with their conflict of interest policy.

The remaining authors declare that the research was conducted in the absence of any commercial or financial relationships that could be constructed as a potential conflict of interest

## Publisher’s note

All claims expressed in this article are solely those of the authors and do not necessarily represent those of their affiliated organizations, or those of the publisher, the editors and the reviewers. Any product that may be evaluated in this article, or claim that may be made by its manufacturer, is not guaranteed or endorsed by the publisher.
