# Supplementary material for: Engineered cord blood megakaryocytes evade killing by allogeneic T-cells for refractory thrombocytopenia
Source: Front Immunol. 2022 Sep 20;13:1018047. doi: 10.3389/fimmu.2022.1018047 (PMC9530569; doi:10.3389/fimmu.2022.1018047)
Supplement: Supplementary file 1 [file DataSheet_1.docx]

**Supplementary Methods**

**Animals**

Animal studies were performed in accordance with MD Anderson Institutional Animal Care and Use Committee approved protocols. Six-week-old NOD.Cg-Prkdc SCID IL2rgtm1Wjl/SzJ (NSG) mice were purchased from Jackson Laboratories (Bar Harbor, ME). Mice were allowed to rest for one week prior to use and housed under pathogen-free conditions in micro-isolator cages with acidified, antibiotic-containing water throughout the experimental procedures.

**CB isolation and expansion**

CB and tissue samples were obtained from the healthy mothers of full-term neonates delivered by elective cesarean section following written informed consent under MD Anderson Institutional Review Board-approved protocols. CB and cord tissues were transported to our Clinical Cell Therapy Laboratory in PlasmaLyte-A with penicillin/streptomycin. Single cell suspensions were prepared by digesting the cord tissue in homogenizers with collagenase, followed by culturing the cells in α-MEM media supplemented with 5% human platelet lysate, 1% L-glutamine, 1% streptomycin-penicillin, and 2UI/mL heparin for 15-20 days. Healthy adult BM-derived MSCs obtained from the MD Anderson MSC Bank were used to support the human CD34+ cell expansion and MK differentiation.

CB mononuclear cells (MNCs) were separated by Ficoll-Hypaque (Sigma Aldrich, St. Louis, MO)-based density gradient centrifugation separation. Whole CB was diluted with an equal amount of PBS and layered over Ficoll-Hypaque solution in 50 ml falcon tubes. MNCs were collected from the middle buffy coat and washed twice with PBS (0.5% human serum albumin) at 400xg for 5min. Approximately 1-3 x10^6^ CD34+ cells were then obtained from the CB MNCs using a positive selection method with CD34+ magnetic beads (#130-046-702, Milteny Biotec, GmbH, Germany), following the manufacturer’s protocol. The purity of CD34+ cells was >90%.

**Flow cytometric analysis**

Flow cytometric analyses were performed with a Fortessa X-20 Cell Analyzer (BD Biosciences, Paolo Alto, CA) and interpreted with Flowjo 10.6.1 version software (Ashland, OR). The following antibodies were used in flow panels in various experiments: BV421 anti-hCD41 (clone MWReg30), APC anti-hCD42b (clone HIP1), A700 anti-hCD61 (clone VI-PL2), PE/Cy7 anti-hCD62P (clone AK4), APC/Cy7 anti-mCD45 (clone S18009F), PE anti-hCD45 (clone 567111), APC/Cy7 anti-mCD45 (S18009F) PE/TR anti-TER119 (cloneTER119), FITC anti-human lineage markers cocktail (CD3,CD11b,CD14,CD16, CD19, CD20,CD235a, CD56), APC anti-hCD34 (clone 581), PE/Cy7 anti-hCD38 (cloneHIT2), BV711 anti-hCD45RA (clone 5H9), PE/Cy5 anti-hCD90 (clone 5E10), PE/Texas Red anti-hCD123 (clone 7G3) and FITC anti-human cutaneous lymphocyte antigen (clone HECA452). All antibodies were purchased from BD Biosciences (San Jose, CA) or BioLegend (San Diego, CA). The detailed antibodies list is given in Supplementary Table 1.

**Imaging flow cytometry**

Imaging flow cytometry (Amnis Image Stream Mark II) analysis was performed to assess the platelet surface expression of CD41a and cytoplasmic alpha tubulin in platelets. Briefly, 2x10^6^ CB-MK-derived platelets were stained with CD41a-BV421 (BioLegend), followed by fixation with 4% paraformaldehyde, permeabilization, blocking with 1% BSA for 1hour, and intracellular staining with FITC-conjugated alpha-tubulin (MA1-19581,1:200 dilution, Thermo Fisher Scientific). Images were analyzed with the IDEAS 6.0 software (Miltenyi Biotech).

**Platelet and mean platelet volume quantification**

Platelets were quantified by centrifuging the cell-containing culture media at 400 x g for 5 min to remove the MKs, followed by staining for platelets with calcein AM (Thermo Fisher) and counting with an automated hemocytometer (Nexcelom Bioscience, Lawrence, MA). Platelets were also identified by flow cytometry as cells 2-7 µm in diameter and expressing CD41 and CD42, while any contaminating CB-MKs were larger (10-35 µm). For select in-vitro experiments, shear stress was applied for 6 hours with a horizontal shaker, followed by vertical vortexing, to liberate platelets prior to MK separation and quantification of platelets.

In some *in-vitro* experiments, platelet count and mean platelet volume were determined using a Sysmex XN-1000 with XN-10 analyzer. For each test, 1 ml sample from each culture condition was transferred to a new 5 ml tube and analyzed by automatic count Sysmex. Sysmex analyzers use the DC sheath flow detection method to count platelets and determine their volume. Briefly, a portion of sample is mixed with the diluent in a pre-set ratio and sent to the detection chamber and passed through the aperture attached with electrodes on each side. The direct current resistance between the electrodes changes as the cells pass through the aperture, causing an electrical pulse change that is proportional to the size of the cell. As soon as the cells have passed the orifice, they are led to the drain. This prevents renewed circulation and a change in the platelet count. The electrical data are converted into graphical displays of volume distribution curves and histograms.

**Human MK colony-forming unit (CFU) assay**

CB MK clonogenic assays were performed using a MegaCult-C (Stem Cell Technologies, Cambridge, MA) kit. Briefly, 1x10^3^ CD34+ cells were seeded in triplicate in 0.9% methylcellulose containing media with recombinant human TPO (50 ng/ml) in serum-free conditions and incubated for 10 to 12 days at 37°C in a fully humidified atmosphere with 5% CO_2_. After culture, cells in double chamber slides were dehydrated and fixed in a methanol–acetone solution at room temperature for 30 min. MK colony staining was performed with a MegaCult CFU-MK kit (Stem Cell Technologies) protocol and stained with anti-human GPIIb/IIIa antibody (#04962, Stem Cell Technologies). Imaging was completed with a light microscope (Zeiss Axio Vert. A1, White Plains, NY) to identify MK-CFUs and proplatelet formation. Giemsa staining and light microscopy image analysis of cytospun cells was also performed to identify polyploid nuclei in mature MKs.

**MK ploidy analyses**

1x10^6^ day 20-25 normal and ROCK inhibitor-treated expanded CB MKs were harvested, washed twice with 5 ml phosphate buffered saline (PBS, without Ca^+2^ or Mg^+2^) and centrifuged at 400 x g for 5 min. The cells were stained with anti-hCD41-BV421, anti-hCD42-APC (BioLegend, San Diego, CA) and fixable aqua dead cell viability dye for 30 min, followed by washing with PBS at 400 x g for 5 min. The cells were fixed with 4% paraformaldehyde for 20 min at room temperature and washed twice with PBS (0.5% BSA). The cells were permeabilized by adding 500 μl 70% chilled ethanol dropwise slowly while vortexing to minimize cell clumping and kept at -20°C for 2-4 hours. The cells were washed with PBS twice and suspended in RNase A solution (Sigma Aldrich, MO) for 1 hour at 4°C to remove the nonspecific propidium iodide (PI) binding to RNA. Finally, the cell pellet was resuspended in 500 μl of FxCycle™ PI/RNase Staining Solution and stained for 30 min at room temperature. The cells were analyzed directly without washing by flow cytometry in the CD41+/CD42+ population.

**Platelet aggregation and activation assays**

The MK-derived platelets from the day 20-25 culture product were harvested and centrifuged at 400 x g for 5 min to remove MKs and residual MSCs. CB platelets were isolated from the MK-free culture suspension by centrifugation at 800 x g for 15 min. The platelets were resuspended in Tyrode’s buffer supplemented with 5 mM glucose, BSA (3 mg/ml) and 1μM prostaglandin E1 (PGE1) to prevent pre-stimulation platelet activation. PGE1 was removed by washing with PBS prior to stimulation and the platelets were resuspended in Tyrode’s buffer (1–3 x 10^8^ platelets/ml) and stimulated with 100 nM or 50 μM of thrombin receptor activating peptide (TRAP-6) for 20 min. The activation status of platelets was studied by measuring the percentage of P-selectin (CD62P) positive cells within CD41+/CD42+ population after stimulation with TRAP-6, using flow cytometry.

For platelet aggregation visualization experiments, the CB-MK-derived human platelets were collected from day 23-25 differentiated MKs culture suspension by centrifugation at 800 x g for 15 min and resuspended in PBS (1% HSA) with 2 mM CaCl_2_. Platelets were stimulated with 5 µg/mL collagen for 5 min at 37°C and visualized for aggregates formation in the tube.

For platelet aggregation quantification experiments, *in-vitro* generated platelets in 250µl serum-free media supplemented with 250μM CaCl_2_ and 50μM MgCl_2_ were stimulated with collagen (5 µg/mL; Bio/Data Corporation). Aggregation was monitored in an optical aggregometer (PAP-8E aggregometer, Bio/Data Corporation, Horsham, PA, USA) for 15-20 min at 37°C with continuous stirring (1,200 rpm).

**Bleeding studies**

For bleeding experiments of mice treated with control or KD045-treated MKs, anesthetized mice were warmed to dilate the blood vessels, followed by amputation of the distal 10mm segment of the tail with a scalpel and immersing in warm normal saline. The bleeding time was counted until bleeding ceased.

**Transmission electron microscopy (TEM) analysis**

Day 20 expanded CB CD34+ derived MKs and unstimulated or TRAP stimulated platelets were isolated and washed twice with PBS by centrifugation at 400 x g for 5 min (1000 x g for 10 min for platelets). The MKs and platelets were fixed with an electron microscopy grade 4% formaldehyde and glutaraldehyde mixture for at least 1 h at room temperature and processed according to an established protocol^1^.

**Western blot**

3x10^6^ CB MKs (day 18 expansion product, n=3 different cord blood units) were cultured in the presence or absence of various concentrations of Y27632 and KD045 ROCK inhibitors for 24h. The cells were washed with PBS and lysed with 1X RIPA lysis buffer (Thermo Fisher Scientific) containing 10 mM Tris-HCl (pH 7.4), 150 mM NaCl, 1% Nonidet P-40, and 1X protease-phosphatase inhibitor mixture (Thermo Fisher Scientific). 20 μg of purified protein was resolved in 4-12% NuPAGE Bis-Tris protein gels and transferred to nitrocellulose membranes. The membrane was probed overnight at 4°C with rabbit anti-phospho LIMK(Thr508 phosphorylation, 1:1000 dilution), rabbit anti- phospho MYPT1(Thr696 phosphorylation,1:1000), and anti-β-actin (1:2000) antibodies. Following incubation, the membrane was washed three times in 1X TBST buffer and stained with horseradish peroxidase (HRP)-conjugated anti-rabbit secondary antibody (1:5000) for 1h at room temperature. Membranes were analyzed using enhanced chemiluminescence-based Super Signal West Dura Extended Duration HRP Substrate (Thermo Fisher Scientific). Target proteins expression relative to β-actin was quantified using Image J software (NIH, Bethesda). All antibodies were purchased from Cell Signaling Technology (Danvers, MA).

**T cell-mediated apoptosis and cytotoxicity assays**

Day 25, fully differentiated control and β2M KO MKs were cultured with preactivated day 14 expanded allogenic T cells from different CB donors for 4h in a 2:1(effector: target) ratio in RPMI media supplemented with 10% FBS,1% L-glutamine, 1% penicillin-streptomycin and recombinant hIL-2 (200 U/ml, R&D Systems, Minneapolis, MN) in U-bottom 96 well plates. Following incubation, the cells were stained for 30 min with fluorophore-conjugated antibodies against hCD42, hCD41, hCD3 and washed with PBS. The cells were stained with 5 μl annexin V-PE (BioLegend) and DAPI (0.5μg/ml) for 30 min in 1X annexin binding buffer (BD Biosciences). The cells were washed and resuspended in 1X binding buffer. The apoptosis comparison was performed in the CD3-/CD41+/CD42+ MKs from control and β2M KO groups by analyzing annexin V+/DAPI+ cells. Chromium-51 (^51^Cr, PerkinElmer, Boston, MA) cytotoxicity release assays were also performed in the ^51^Cr prelabeled control and β2M KO MKs by coculturing with activated allogenic T cells (effector: target of 20:1,10:1, 5:1 and 1:1) for 4h in V-bottom 96 well plates. The percentage cell lysis/killing was determined by quantifying the released ^51^Cr from the cocultures (mean of triplicate samples) using a Lumaplate^TM^ reader. The negative control was MKs alone and the positive control was MKs treated with 20% SDS.

### **CRISPR/Cas9-mediated β2M KO**

CB-derived CD34+ cells were electroporated with CRISPR/Cas9 complexes on day 3 of MK differentiation using the predesigned Alt-R CRISPR/Cas9 CRISPR RNAs (crRNA) with >90% on-target score, which was purchased from Integrated DNA Technologies (IDT, Coralville, IA). 2μM crRNAs with the highest cutting efficiency were chosen from up to 3 crRNAs screened for the β2M (exon 2) gene target. The crRNA sequence AAGTCAACTTCAATGTCGGA (PAM sequence-TGG), targeting exon 2 of β2M gene, was selected based on the best efficiency and was used for the final KO experiments. To induce the CRISPR-Cas9 mediated KO, the crRNA+ tracrRNA duplexes were prepared by incubation at 95°C for 5 min in a thermocycler at equimolar concentrations. High-fidelity Cas9 protein (IDT, Coralville, IA) and sgRNA (crRNA-tracrRNA combination) were incubated at room temperature for 15 min in a 1:1 ratio. The CD34+ cells were electroporated with the incubation product (RNAs hybrid and Cas9 complex) using the Neon Transfection System (Thermo Fisher Scientific) under optimized electroporation conditions (1600V for 10 msec with 3 pulses in resuspension buffer T). The electroporated cells were analyzed by flow cytometry for surface β2M expression in CD34+ and differentiating MK precursors at 72h to validate the KO efficiency. Following successful KO confirmation, the CD34+/MKs were cultured in the standard MK differentiation conditions mentioned earlier.

**Supplementary References**

1. Graham L, Orenstein JM. Processing tissue and cells for transmission electron microscopy in diagnostic pathology and research. *Nature Protocols*. 2007;2(10):2439-2450.

**Supplementary Table 1. Reagents used in this study.**

| **Antibody** | **Cat Number** | **Clone** | **Source** | **Dilution/**  **Concentration** |
| --- | --- | --- | --- | --- |
| Brilliant Violet 421™ anti-human CD41 Antibody | 303730 | HIP8 | Biolegend | 1;50 |
| PE/Cyanine7 anti-human CD62P (P-Selectin) Antibody | 304922 | AK4 | Biolegend | 1:25 |
| Alexa Fluor® 647 anti-human CD42b Antibody | 303924 | HIP1 | Biolegend | 1;50 |
| PE/Dazzle™ 594 anti-mouse TER-119/Erythroid Cells Antibody | 116244 | TER119 | Biolegend | 1;50 |
| Alexa Fluor® 700 anti-human CD61 Antibody | 336420 | VI-PL2 | Biolegend | 1;25 |
| PE Rat Anti-Mouse CD45 | I3/2.3 | 567111 | BD Bioscience | 1;20 |
| Alexa Fluor® 488 anti-mouse CD41 Antibody | 133908 | MWReg30 | Biolegend | 1;50 |
| Alexa Fluor® 647 anti-mouse CD41 Antibody | 133934 | MWReg30 | Biolegend | 1;50 |
| APC/Cyanine7 anti-mouse CD45 Recombinant Antibody | 157204 | S18009F | BD Bioscience | 1;20 |
| PE Annexin V | 640908 |  | Biolegend | 1;50 |
| 4′,6-diamidino-2-phenylindole(DAPI) | 62248 |  | Thermo Fisher | 1µg/ml |
| Goat anti-Rabbit IgG (H+L), Superclonal™ Recombinant Secondary Antibody, Alexa Fluor 647 | A27040 |  | Thermo Fisher | 1;200 |
| F(ab')2-Goat anti-Rabbit IgG (H+L) Cross-Adsorbed Secondary Antibody, PE | 31864 |  | Thermo Fisher | 1;200 |
| Phospho-MYPT1 (Thr696) Antibody | 5163S |  | Cell Signaling Technology | 1;1000 |
| Phospho-LIMK1 (Thr508)/LIMK2 (Thr505) Antibody | 3841S |  | Cell Signaling Technology | 1;1000 |
| MYPT1 Rabbit Antibody | 8574S |  | Cell Signaling Technology | 1;1000 |
| LIVE/DEAD™ Fixable Aqua Dead Cell Stain Kit, for 405 nm excitation | L34966 |  | Thermo Fisher Scientific | 1µg/ml |
| FxCycle™ PI/RNase Staining Solution | F10797 |  | Thermo Fisher Scientific | 500µl |
| Ribonuclease A from bovine pancreas | 10109169001 |  | Sigma Aldrich | as recommended |
| β-Actin (D6A8) Rabbit mAb (HRP Conjugate) | 12620S |  | Cell Signaling Technology | 1;2000 |
| Anti-rabbit IgG, HRP-linked Antibody | 7074P2 |  | Cell Signaling Technology | 1;5000 |
| Prostaglandin E_1_ | P5515-5MG |  | Sigma Aldrich | 1µM |
| Y-27632 | S1049 |  | Selleckchem | 1-10µM |
| KD045 |  |  | Kadmon Corporation | 100nM-10µM |
| CellTrace™ CFSE Cell Proliferation Kit, for flow cytometry | C34554 |  | Thermo Fisher Scientific | 2µM |
| Annexin V Binding Buffer | 422201 |  | Biolegend | 1X |


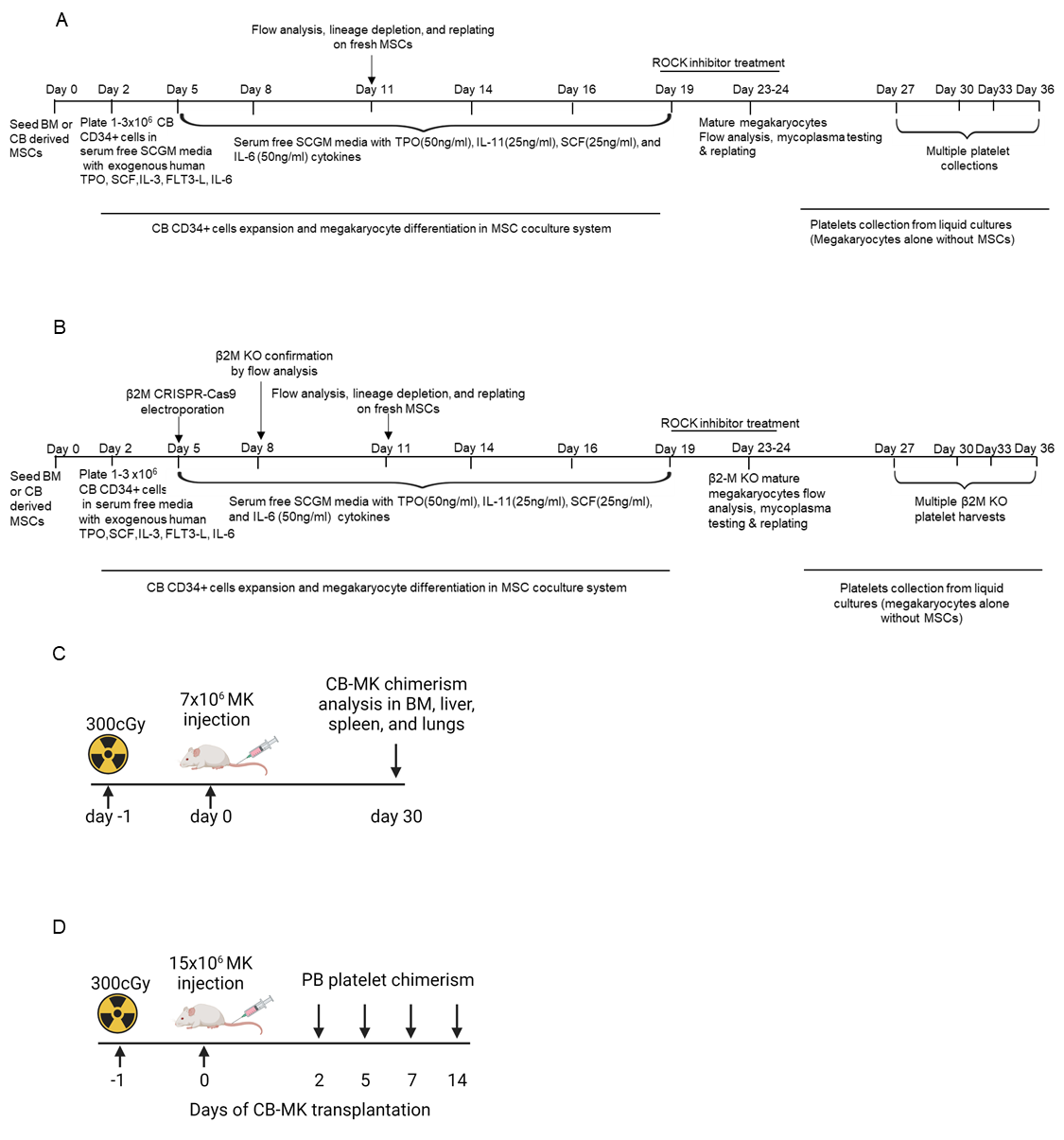


**Supplementary Figure 1**: **MK differentiation strategy from CB-derived HSCs and generation of CRISPR-Cas9 engineered β2M deficient cells.**

(A) Schematic of the detailed strategy of MK and platelet production from CB-derived CD34+ cells in an MSC coculture system supplemented with a human recombinant cytokine cocktail and pharmacological ROCK inhibition. (B) Schematic of CRISPR-Cas9-mediated β2M KO in HSCs, followed by MK differentiation/expansion to produce β2M deficient mature MKs and platelets. (C) Schematic of the strategy for analyzing CB-MK chimerism in the bone marrow (BM), liver, spleen, and lungs of sub lethally irradiated mice *in-vivo* following transfer of CB-MKs. (D) Schematic of the strategy for analyzing peripheral blood (PB) platelet chimerism in sub lethally irradiated mice *in-vivo* following transfer of MKs.


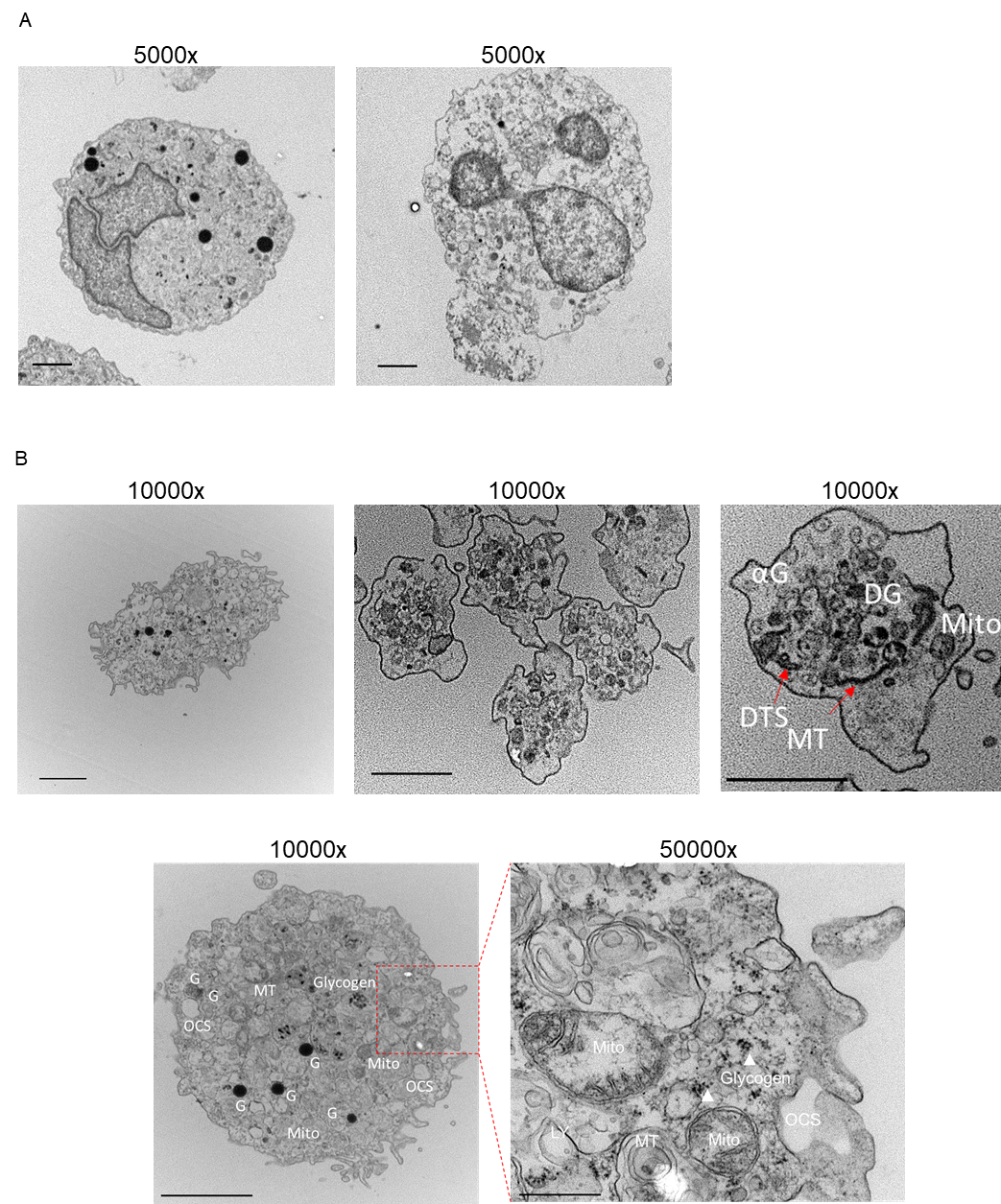


**Supplementary Figure 2:** **Transmission electron microscopy (TEM) of CB-derived MKs and platelets.** (A) Example TEM 5000X (scale bar = 2µm) magnification images of CB-derived MKs. (B) Example TEM 10000X (scale bar = 2µm) and 50000X (scale bar = 500nm) magnification images of platelets generated from CB-derived MKs (Abbreviations: αG, alpha granule; DG, dense granule; DTS, dense tubular system; G, granule; Ly, lysosome; Mito, mitochondria; MT, microtubule; OCS, open canalicular system).


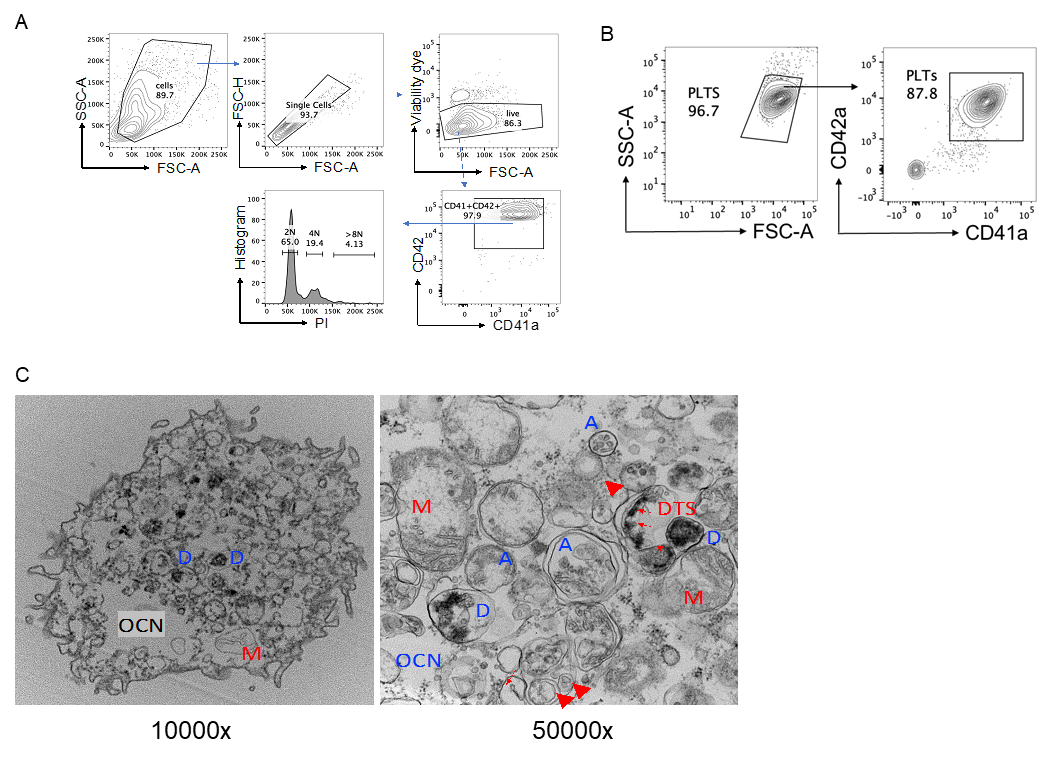


**Supplementary Figure 3: Propidium iodide and transmission electron microscopy (TEM) indicate characteristics of CB-derived MKs and platelets**.

(A) Flow cytometry based propidium iodide staining histogram plot to study the percentage polyploidy in the CD41+CD42+ CB-MKs. (B) Flow cytometry gating strategy for CD41a+CD42a+ platelets. (C) transmission electron microscopy (TEM) 10000X (left) and 50000X (right) magnification images of a CB-MK-derived platelet (Abbreviations: A, alpha granule; D, dense granule; DTS, dense tubule system; M, mitochondria; OCN, open canalicular network).

**
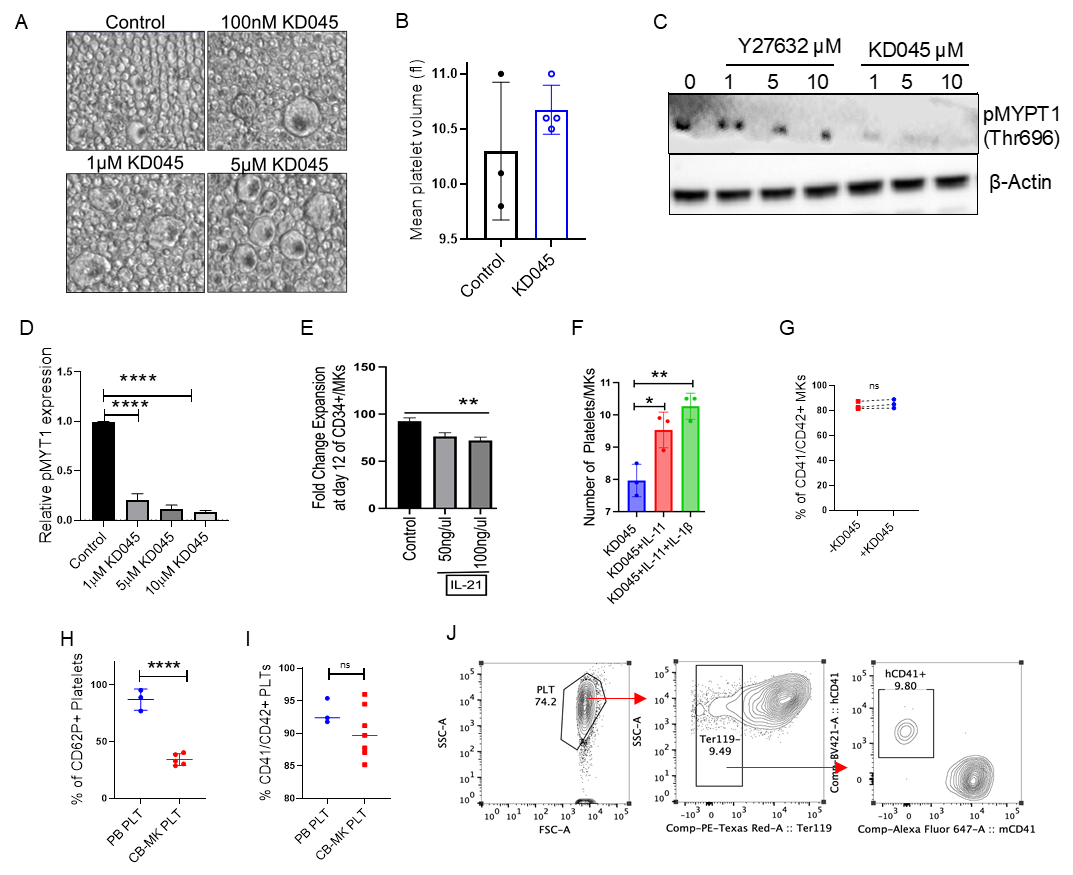
**

**Supplementary Figure 4: KD045 accelerates MK maturation through ROCK inhibition.**

(A) 10X bright field images of day 18 cultured untreated (control) and KD045-treated MKs exhibiting increased cell sizes in KD045-treated groups. (B) Mean platelet volume of platelets generated from untreated or KD045-treated CB-MKs (n=3-4 per group). (C) Western blot of pMYPT1(Thr696) expression in control, KD045, or Y27632-treated MKs after 24h (n=3 cords). (D) pMYPT1(Thr696) relative (β-actin normalized) expression in control and KD045-treated MKs (n=3, ****p<0.0001). (E) Fold change expansion of untreated and IL-21-treated MKs (n=2-3, p=0.0082). (F) Number of platelets generated per MK after treatment with KD045 alone, or KD045 and IL-11 (25ng/ml), or KD045, IL-11, and IL-1β (20ng/ml) (n=3, *p<0.05, **p<0.01). (G) Percentage of CB-MKs expressing CD41/CD42 after culture in the presence or absence of KD045 (n=3). (H) Percentage of peripheral blood (PB) and CB-MK-derived platelets expressing CD62P (n=3-5, ****p<0.0001). (I) Percentage of PB and CB-MK-derived platelets expressing CD41/CD42 (n=3-7). (J) Gating strategy to analyze the percentage chimerism of hCD41+ CB MK-derived platelets in NSG mouse blood.

**Supplementary Figure 5: Sub-lethal irradiation-induced thrombocytopenia in NSG mice.**

Platelet counts over time in the peripheral blood of NSG mice following 300cGy irradiation on day 0.

**
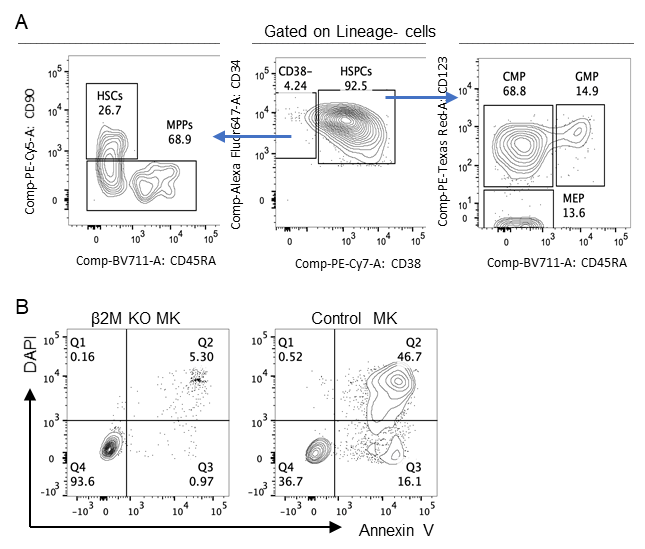
**

**Supplementary Figure 6.**

(A) Gating strategy to identify the various CB-derived hematopoietic stem/progenitors’ populations. (B) Flow cytometry-based contour plots depicting annexin V and DAPI staining in Cas9 control and β2M KO MKs.

**
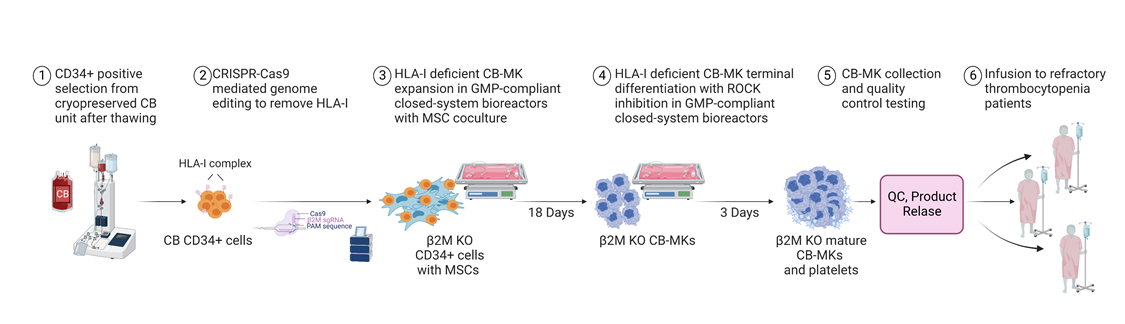
**

**Supplementary Figure 7.** **Overview of the proposed GMP-compliant strategy to generate CB-MKs and platelets.**
